# Weekend admission and outcomes in cancer-associated pulmonary embolism: a cross-sectional national inpatient sample study, 2016–2022

**DOI:** 10.1038/s41598-026-50298-4

**Published:** 2026-05-22

**Authors:** Xiaoyi Zhang, Na Xiao, Boon Jian San, Simo Du, Zizhuo Yang, Cho-Hao Lee

**Affiliations:** 1 Jacobi Medical Center, Department of Medicine, Bronx, NY USA; 2 Cheeloo College of Medicine, Shandong University, Shandong, China; 3 Department of Oncology, Memorial Sloan Kettering Cancer Center, Manhattan, NY USA; 4 Division of Hematology and Oncology, Department of Internal Medicine, Tri- Service General Hospital, National Defense Medical University, Taipei, Taiwan

**Keywords:** Pulmonary embolism, Venous thromboembolism, Malignancy, Cancer-associated thrombosis, Weekend effect, In-hospital mortality, National inpatient sample, Cancer, Diseases, Health care, Medical research, Oncology, Risk factors

## Abstract

The “weekend effect” has been variably associated with outcomes in acute pulmonary embolism (PE), but its relevance in cancer-associated PE remains uncertain. Among U.S. hospitalizations for cancer-associated PE, is weekend admission independently associated with in-hospital mortality, and do PE-related interventions differ by day of admission? Retrospective cross-sectional study using the HCUP National Inpatient Sample (2016–2022). Adults (≥ 18 years) hospitalized with a principal diagnosis of PE and concomitant malignancy were included. Weekend admission was Saturday–Sunday. Survey-weighted analyses in R generated national estimates. Survey-weighted logistic regression estimated adjusted odds ratios (ORs) for in-hospital mortality. We compared PE-related therapies/procedures (mechanical ventilation, vasopressors, systemic thrombolysis, and inferior vena cava [IVC] filter placement), including hospital-day-1 use (0 days to procedure). We identified 37,491 unweighted cancer-associated PE hospitalizations, representing approximately 187,455 weighted national hospitalizations, of which 7,804 unweighted hospitalizations (20.8%) were weekend admissions. Weekend admissions had higher acuity, with the administrative marker of high-acuity PE more frequent on weekends (29.1% vs. 26.3%), and higher unadjusted mortality (6.6% vs. 5.9%). In the fully adjusted model (*N* = 36,408; deaths = 2,210), weekend admission was not associated with mortality (adjusted OR 1.04, 95% CI 0.93–1.16). Mechanical ventilation was more frequent on weekends (4.7% vs. 3.6%). Thrombolysis and overall IVC filter use were similar, but hospital-day-1 IVC filter placement was less common on weekends (14.7% vs. 18.9%). Higher unadjusted weekend mortality in cancer-associated PE appears largely explained by case mix and severity rather than weekend admission itself.

**Supplementary Information:**

The online version contains supplementary material available at 10.1038/s41598-026-50298-4.

## Introduction

Pulmonary embolism (PE) is a common and potentially fatal complication in patients with malignancy. Cancer-related hypercoagulability and treatment exposures increase venous thromboembolism risk, and PE in this population is associated with substantial acute morbidity, frequent critical care needs, and high short-term mortality^1^. In both classic and contemporary literature, cancer-associated thrombosis has been consistently linked to worse prognosis, and PE has been described as a leading noncancer cause of death among patients with malignancy^2^. In administrative data focused on hospitalized PE, patients with cancer have higher in-hospital mortality and greater resource utilization than those without cancer, underscoring the importance of optimizing acute PE care pathways in oncologic populations^3,4^.

The “weekend effect”, worse outcomes among hospitalizations admitted on weekends compared with weekdays, has been reported across multiple acute conditions but remains inconsistently observed and variably explained^5^. In a seminal study of PE hospitalizations, Nanchal and colleagues reported higher mortality for weekend admissions and observed differences in the timeliness of care processes, including later placement of inferior vena cava (IVC) filters^6^. However, subsequent investigations across conditions have suggested that observed weekend-associated mortality differences may attenuate after accounting for differences in acuity at presentation, admission type, and case mix—factors that are particularly relevant in oncology, where physiologic reserve, metastatic burden, and competing risks can dominate outcomes^5^.

Whether a weekend effect persists specifically among cancer-associated PE hospitalizations in the modern era is therefore uncertain. Compared with general PE populations, cancer-associated PE represents a distinct clinical phenotype with higher baseline vulnerability, greater heterogeneity by primary cancer type and metastatic status, and frequent concomitant complications that may influence both treatment decisions (e.g., thrombolysis eligibility) and outcomes^7^. From a systems perspective, weekend resource constraints, real or perceived, could plausibly affect acute PE care processes such as escalation to mechanical ventilation, vasopressor initiation, thrombolysis pathways, and procedural decision-making, yet administrative datasets have rarely evaluated these pathways in contemporary cancer-associated PE cohorts^2,8^.

Accordingly, we used the HCUP National Inpatient Sample (2016–2022) to evaluate the association between weekend admission and in-hospital mortality among U.S. hospitalizations for cancer-associated PE. We further assessed (1) whether any observed weekend–mortality association was explained by differences in illness severity and clinical risk factors using sequential adjustment, (2) heterogeneity of the weekend association across key hospitalization-level and hospital-level subgroups, (3) cancer-type–specific mortality and weekend effects, and (4) differences in PE-related interventions and their timing by day of admission^9^. We hypothesized that weekend admission would be associated with higher in-hospital mortality and differences in PE-related interventions and their timing compared with weekday admissions, and that any crude weekend association would attenuate after adjustment for illness severity and case mix.

## Materials and methods

### Data source and study design

We conducted a retrospective cross-sectional study using the Healthcare Cost and Utilization Project (HCUP) National Inpatient Sample (NIS) from January 1, 2016 through December 31, 2022. The NIS is a nationally representative, stratified sample of U.S. community hospital discharges and includes discharge-level sampling weights and design variables (strata and clustering) to enable national estimates. Because the NIS is a discharge-level dataset, analyses reflect hospitalizations rather than unique patients.

*Ethics approval and informed consent.* This study is a secondary analysis of the Healthcare Cost and Utilization Project (HCUP) National Inpatient Sample (NIS), which contains de-identified discharge-level data and no direct patient identifiers. All methods were carried out in accordance with relevant guidelines and regulations and in compliance with the HCUP Data Use Agreement (DUA). The need to obtain ethical approval was waived by the Einstein Institutional Review Board (IRB), Albert Einstein College of Medicine. Due to the retrospective nature of the study, the Einstein Institutional Review Board (IRB), Albert Einstein College of Medicine, waived the need for obtaining informed consent.

### Study population

We identified adult hospitalizations (age ≥ 18 years) with a principal discharge diagnosis of pulmonary embolism (PE) using International Classification of Diseases, Tenth Revision, Clinical Modification (ICD-10-CM) codes (code list provided in the code list in eTable S1). Cancer-associated PE hospitalizations were defined by the presence of ICD-10-CM codes for solid or hematologic malignancy during the index admission; primary cancer type categories were assigned using a prespecified coding algorithm (eTable S2). Metastatic disease was identified using ICD-10-CM codes for secondary malignant neoplasms (eTable S2). The study size was determined by inclusion of all eligible NIS hospitalizations meeting the above criteria during 2016–2022 (no a priori sample-size calculation). Absolute numbers reported in the text and tables represent unweighted hospitalization counts, whereas percentages, national estimates, and regression analyses were derived using the NIS survey design and discharge weights.

### Definition of independent variables

Weekend admission was defined as admissions occurring between 12:01 am Saturday and 11:59 pm Sunday, with all other admissions classified as weekday admissions. Covariates recorded at the hospitalization level included demographics (age group, sex, race/ethnicity), primary payer, calendar year, cancer type, metastatic disease, and comorbidities identified using ICD-10-CM codes (eTable S2, S3). Hospital characteristics included region, bed size, and location/teaching status as defined by HCUP. Age was modeled categorically using prespecified strata (< 65 years and ≥ 65 years) consistent with subgroup analyses.

We classified PE using a prespecified administrative-data severity marker intended to capture high-acuity PE presentations, defined by the presence of diagnosis and/or procedure indicators for advanced supportive therapies early in the hospitalization (eTable S4). This administrative severity marker does not correspond directly to guideline-based PE risk classification.

### Outcomes

The primary outcome was in-hospital mortality. Secondary outcomes included length of stay and total hospital charges (analyzed among survivors), discharge disposition, and PE-related therapies/procedures including mechanical ventilation, vasopressor use, systemic thrombolysis, and inferior vena cava (IVC) filter placement.

Because the NIS does not include procedure time but does include procedure day, we evaluated time to procedure in hospital days (e.g., “hospital day 1” corresponds to 0 days to procedure). We compared both overall use and first-hospital-day use of thrombolysis and IVC filter placement across weekend versus weekday admissions. First-hospital-day use was analyzed as a timing descriptor and should not be interpreted as a direct measure of appropriateness or quality of care.

### Statistical analysis

All analyses incorporated the NIS complex survey design to produce nationally representative estimates. We used R (R Foundation for Statistical Computing) and the survey package to specify survey design elements (discharge weights, stratification, and clustering). Baseline characteristics were compared using survey-weighted χ² tests for categorical variables and survey-weighted t tests for continuous variables; Wilcoxon rank-sum tests were used when appropriate. Because length of stay and hospital charges were right-skewed, we analyzed log-transformed values using survey-weighted linear regression.

To estimate the independent association between weekend admission and in-hospital mortality, we fit survey-weighted logistic regression models and report adjusted odds ratios (ORs) with 95% confidence intervals. We performed sequential model building to assess attenuation of the weekend association with progressive adjustment for demographics, payer, hospital characteristics, the administrative marker of high-acuity PE, cancer type, metastatic disease, and comorbidities. Prespecified subgroup analyses evaluated the weekend effect within strata (e.g., age, sex, race/ethnicity, payer, hospital characteristics, the administrative marker of high-acuity PE, metastatic disease, cancer type, and year) using models adjusted for age, sex, and year. Cancer-type–specific weekend effects were estimated using models adjusted for age, sex, and the administrative marker of high-acuity PE; adjusted estimates were suppressed in strata with sparse events. Additional methodological details are provided in the Supplementary eMethods.

### Missing data and bias considerations

We used survey-weighted complete-case analysis for multivariable models by excluding hospitalizations with missing values in prespecified adjustment covariates required for the fully adjusted model. To mitigate confounding, we used sequential model building and included markers of acute severity (the administrative marker of high-acuity PE), cancer burden (metastatic disease and cancer type), hospitalization-level sociodemographic characteristics, payer, hospital characteristics, and comorbidities. We recognize potential bias from diagnostic/procedure coding misclassification and residual confounding due to unavailable physiologic and imaging variables; these limitations are addressed in the Discussion. This study is reported in accordance with the STROBE guideline for cross-sectional studies.

## Results

### Baseline characteristics

We identified 37,491 unweighted cancer-associated PE hospitalizations from 2016 to 2022, representing approximately 187,455 weighted national hospitalizations. Of these, 29,687 unweighted hospitalizations (79.2%) were weekday admissions and 7,804 (20.8%) were weekend admissions. Overall, demographic and hospital characteristics were broadly similar between groups, although several clinically meaningful differences were observed.

Weekend admissions reflected a higher-acuity case mix: the administrative severity marker for high-acuity PE was more frequent on weekends (29.1% vs. 26.3%, *P* < 0.001), and weekend hospitalizations were less likely to be elective (2.0% vs. 3.1%, *P* < 0.001). Age distribution differed modestly (*P* = 0.006) despite a similar mean age (67.1 years in both groups). Geographic region also varied (*P* = 0.003), with a slightly higher proportion of weekend admissions in the West (18.8% vs. 17.0%).

Cancer characteristics were generally comparable, but primary cancer type distribution differed (*P* = 0.007), including a higher proportion of hematologic malignancies among weekend admissions (12.3% vs. 10.9%). Metastatic disease prevalence was high in both groups and similar (48.5% weekend vs. 49.7% weekday, *P* = 0.065). In unadjusted analyses, in-hospital mortality was higher for weekend admissions (6.6% vs. 5.9%, *P* = 0.026) (Table 1).

**Table 1:** Baseline characteristics of hospitalizations for cancer-associated pulmonary embolism by admission day (weekday vs. weekend), NIS 2016–2022. Absolute counts are unweighted numbers of hospitalizations; percentages are survey-weighted.

Characteristic	Total (*N* = 37,491)	Weekday (*n* = 29,687)	Weekend (*n* = 7,804)	*P* value
DEMOGRAPHICS				
**Age group**,** n (%)**				0.006
18–34 years	622 (1.7)	480 (1.6)	142 (1.8)	
35–49 years	2,551 (6.8)	1,990 (6.7)	561 (7.2)	
50–64 years	11,313 (30.2)	8,950 (30.1)	2,363 (30.3)	
65–79 years	16,871 (45.0)	13,483 (45.4)	3,388 (43.4)	
≥80 years	6,134 (16.4)	4,784 (16.1)	1,350 (17.3)	
**Age**,** mean (SD)**	67.1 (12.7)	67.1 (12.6)	67.1 (13.1)	0.95
**Sex**,** n (%)**				0.467
Female	19,525 (52.1)	15,490 (52.2)	4,035 (51.7)	
Race, n (%)				0.072
White	26,809 (71.5)	21,317 (71.8)	5,492 (70.4)	
Black	5,993 (16.0)	4,667 (15.7)	1,326 (17.0)	
Hispanic	2,221 (5.9)	1,752 (5.9)	469 (6.0)	
Asian/ Pacific Islander	574 (1.5)	443 (1.5)	131 (1.7)	
Native American	127 (0.3)	97 (0.3)	30 (0.4)	
Other	785 (2.1)	623 (2.1)	162 (2.1)	
**Primary payer**,** n (%)**				0.216
Medicare	22,198 (59.2)	17,551 (59.1)	4,647 (59.5)	
Medicaid	3,605 (9.6)	2,804 (9.4)	801 (10.3)	
Private	10,033 (26.8)	7,999 (26.9)	2,034 (26.1)	
Self-pay	661 (1.8)	534 (1.8)	127 (1.6)	
No charge	59 (0.2)	47 (0.2)	12 (0.2)	
Other	895 (2.4)	719 (2.4)	176 (2.3)	
**Teaching status**				0.543
Rural non-teaching	2,898 (7.7)	2,288 (7.7)	610 (7.8)	
Urban non-teaching	6,866 (18.3)	5,470 (18.4)	1,396 (17.9)	
Urban teaching	27,727 (74.0)	21,929 (73.9)	5,798 (74.3)	
**Hospital location**				0.421
Large central metro	10,241 (27.3)	8,057 (27.1)	2,184 (28.0)	
Large fringe metro	9,709 (25.9)	7,755 (26.1)	1,954 (25.0)	
Medium metro	7,845 (20.9)	6,222 (21.0)	1,623 (20.8)	
Small metro	3,582 (9.6)	2,832 (9.5)	750 (9.6)	
Micropolitan	3,505 (9.3)	2,760 (9.3)	745 (9.5)	
Non-core	2,511 (6.7)	1,991 (6.7)	520 (6.7)	
**Bed size**				0.597
Small	7,247 (19.3)	5,710 (19.2)	1,537 (19.7)	
Medium	10,335 (27.6)	8,208 (27.6)	2,127 (27.3)	
Large	19,909 (53.1)	15,769 (53.1)	4,140 (53.0)	
**Region**				0.003
Northeast	7,599 (20.3)	6,062 (20.4)	1,537 (19.7)	
Midwest	9,449 (25.2)	7,515 (25.3)	1,934 (24.8)	
South	13,927 (37.1)	11,059 (37.3)	2,868 (36.8)	
West	6,516 (17.4)	5,051 (17.0)	1,465 (18.8)	
CLINICAL CHARACTERISTICS				
**Admission type**,** n (%)**				< 0.001
Elective	1,076 (2.9)	921 (3.1)	155 (2.0)	
**Administrative marker of high-acuity PE†**,** n (%)**				< 0.001
Yes	10,067 (26.9)	7,795 (26.3)	2,272 (29.1)	
CANCER CHARACTERISTICS				
**Primary cancer type**,** n (%)**				0.007
Other	12,408 (33.1)	9,880 (33.3)	2,528 (32.4)	
Lung	8,523 (22.7)	6,774 (22.8)	1,749 (22.4)	
GI malignancies	4,414 (11.8)	3,516 (11.8)	898 (11.5)	
Hematologic	4,191 (11.2)	3,233 (10.9)	958 (12.3)	
Breast	3,208 (8.6)	2,556 (8.6)	652 (8.4)	
Colorectal	2,683 (7.2)	2,130 (7.2)	553 (7.1)	
Prostate	2,064 (5.5)	1,598 (5.4)	466 (6.0)	
**Metastatic disease**,** n (%)**				0.065
Yes	18,549 (49.5)	14,761 (49.7)	3,788 (48.5)	
OUTCOMES				
**In-hospital mortality**,** n (%)**				0.026
Died	2,284 (6.1)	1,766 (5.9)	518 (6.6)	
**Length of stay**,** days**				0.335
Mean (SD)	5.1 (5.3)	5.1 (5.3)	5.1 (5.3)	
Median (IQR)	4 (2–6)	4 (2–6)	4 (2–6)	
**Total charges**,** $**				0.125
Mean (SD)	58,651 (77,841)	58,352 (79,427)	59,787 (71,481)	

†Administrative severity marker for high-acuity PE, defined by the presence of respiratory failure (J96.0x), cardiogenic shock (R57.0), or cardiac arrest (I46.x). This construct is not equivalent to guideline-based PE severity classification.

### Multivariable predictors of in-hospital mortality

Of 37,491 eligible cancer-associated PE hospitalizations, 1,083 (2.9%) had missing data in one or more prespecified adjustment covariates and were excluded from multivariable models, leaving 36,408 hospitalizations for the fully adjusted analysis. Missingness was most common for race/ethnicity and (less frequently) payer and hospital characteristics. All eligible hospitalizations meeting inclusion criteria were included in descriptive analyses; complete-case restriction was applied only to multivariable regression models requiring fully observed covariates.

In the fully adjusted multivariable logistic regression model (Model 8; *N* = 36,408 hospitalizations; deaths = 2,210), weekend admission was not independently associated with in-hospital mortality (adjusted OR 1.04, 95% CI 0.93–1.16; *P* = 0.531). Model performance was strong, with good discrimination (C-statistic 0.820, 95% CI 0.811–0.829) and acceptable calibration (Hosmer–Lemeshow χ²=15.39, df = 8, *P* = 0.052).

Mortality risk was driven primarily by acute illness severity, cancer burden, and comorbidity profile. The administrative marker of high-acuity PE was the dominant predictor (adjusted OR 6.28, 95% CI 5.70–6.92; *P* < 0.001), and metastatic disease was also strongly associated with higher mortality (adjusted OR 1.81, 95% CI 1.64–2.00; *P* < 0.001). Several comorbidities conferred substantial excess risk, including fluid and electrolyte disorders (OR 2.55, 95% CI 2.32–2.81), arrhythmias (OR 1.74, 95% CI 1.57–1.92), coagulopathy (OR 1.53, 95% CI 1.38–1.70), weight loss (OR 1.41, 95% CI 1.26–1.56), and congestive heart failure (OR 1.32, 95% CI 1.18–1.49) (all *P* < 0.001).

Sociodemographic and system-level factors also showed independent associations. Relative to Medicare, self-pay hospitalizations had higher mortality risk (OR 2.02, 95% CI 1.46–2.74; *P* < 0.001), as did other payer (OR 1.56, 95% CI 1.18–2.03; *P* = 0.001), while Medicaid was not significantly different (OR 1.20, 95% CI 0.98–1.45; *P* = 0.071). Compared with hospitalizations involving White patients, hospitalizations involving Black patients (OR 1.30, 95% CI 1.15–1.48; *P* < 0.001) and hospitalizations involving Asian/Pacific Islander patients (OR 1.83, 95% CI 1.34–2.46; *P* < 0.001) had higher adjusted mortality. At the hospital level, urban teaching hospitals (OR 1.23, 95% CI 1.04–1.48; *P* = 0.021) and large bed-size hospitals (OR 1.24, 95% CI 1.09–1.41; *P* = 0.001) were associated with higher mortality, whereas admissions in the Midwest, South, and West had lower mortality than the Northeast (OR 0.75/0.84/0.86, respectively; all *P* < 0.05).

Cancer type was variably associated with risk: lung cancer was associated with higher mortality (OR 1.14, 95% CI 1.02–1.28; *P* = 0.024), while breast, colorectal, and prostate cancers were associated with lower mortality (OR 0.64/0.78/0.76, respectively; all *P* < 0.05). Age strata and sex were not independently associated with mortality in the final model (Table 2).

To address potential confounding by goals-of-care status, we performed a sensitivity analysis incorporating ICD-10-CM codes for palliative care encounter and DNR status into the fully adjusted model. Weekend admission remained not significantly associated with in-hospital mortality after adjustment for a combined palliative/DNR indicator (OR 1.018, 95% CI 0.902–1.148; *P* = 0.776). Results were similar in a model including palliative care and DNR as separate covariates, in which the weekend-admission estimate remained non-significant (OR 0.992, 95% CI 0.876–1.122; *P* = 0.894). In that model, both palliative care and DNR status were independently associated with higher mortality (OR 3.75, 95% CI 3.314–4.243; and OR 3.713, 95% CI 3.291–4.189, respectively). Results were also similar when hospitalizations with either code were excluded (OR 0.987, 95% CI 0.802–1.215; *P* = 0.903) (eTable S5).

**Table 2:** Multivariate Logistic Regression Analysis of Factors Associated with In-Hospital Mortality.

Variable	OR	95% CI	*P* value
PATIENT DEMOGRAPHICS			
**Weekend admission (vs. weekday)**	1.04	(0.93–1.16)	0.531
**Age group (ref: 18–34 years)**	1.00	Reference	
35–49 years	0.76	(0.50–1.18)	0.209
50–64 years	0.85	(0.58–1.27)	0.404
65–79 years	0.86	(0.58–1.30)	0.452
≥80 years	0.94	(0.63–1.44)	0.755
**Sex (ref: Male)**	1.00	Reference	
Female	0.92	(0.84–1.02)	0.098
**Race (ref: White)**	1.00	Reference	
Black	1.30	(1.15–1.48)	< 0.001
Hispanic	1.19	(0.98–1.44)	0.078
Asian/PI	1.83	(1.34–2.46)	< 0.001
Native American	0.99	(0.42–2.02)	0.981
Other	0.92	(0.65–1.26)	0.614
**Primary payer (ref: Medicare)**	1.00	Reference	
Medicaid	1.20	(0.98–1.45)	0.071
Private	1.01	(0.88–1.17)	0.847
Self-pay	2.02	(1.46–2.74)	< 0.001
No charge	1.38	(0.38–3.78)	0.575
Other	1.56	(1.18–2.03)	0.001
**HOSPITAL CHARACTERISTICS**			
**Teaching status (ref: Rural non-teaching)**			
Rural non-teaching	1.00	Reference	
Urban non-teaching	1.10	(0.91–1.35)	0.337
Urban teaching	1.23	(1.04–1.48)	0.021
**Hospital location (ref: Large central metro)**	1.00	Reference	
Large fringe metro	0.96	(0.85–1.08)	0.477
Medium metro	0.97	(0.85–1.10)	0.654
Small metro	1.05	(0.89–1.24)	0.560
Micropolitan	1.10	(0.92–1.30)	0.303
Non-core	1.14	(0.94–1.37)	0.190
**Bed size (ref: Small)**	1.00	Reference	
Medium	1.05	(0.91–1.21)	0.529
Large	1.24	(1.09–1.41)	0.001
**Region (ref: Northeast)**	1.00	Reference	
Midwest	0.75	(0.65–0.86)	< 0.001
South	0.84	(0.74–0.95)	0.007
West	0.86	(0.74–0.99)	0.041
**CLINICAL CHARACTERISTICS**			
Elective admission (vs. emergency)	1.46	(1.11–1.89)	0.006
Administrative marker of high-acuity PE	6.28	(5.70–6.92)	< 0.001
**CANCER CHARACTERISTICS**			
Lung cancer	1.14	(1.02–1.28)	0.024
Breast cancer	0.64	(0.51–0.78)	< 0.001
Colorectal cancer	0.78	(0.63–0.94)	0.012
Prostate cancer	0.76	(0.60–0.96)	0.023
Pancreatic cancer	1.16	(0.97–1.38)	0.093
Metastatic disease	1.81	(1.64–2.00)	< 0.001
**ELIXHAUSER COMORBIDITIES**			
Fluid and electrolyte disorders	2.55	(2.32–2.81)	< 0.001
Cardiac arrhythmias	1.74	(1.57–1.92)	< 0.001
Coagulopathy	1.53	(1.38–1.70)	< 0.001
Weight loss	1.41	(1.26–1.56)	< 0.001
Congestive heart failure	1.32	(1.18–1.49)	< 0.001
Renal failure	1.02	(0.89–1.16)	0.819
Chronic pulmonary disease	0.99	(0.89–1.10)	0.899
Valvular disease	0.77	(0.63–0.94)	0.010

### Subgroup analyses of the weekend effect on in-hospital mortality

In prespecified subgroup analyses evaluating the “weekend effect” (weekend vs. weekday admission) with odds ratios adjusted for age, sex, and year, the magnitude of association varied across strata. Overall, no statistically significant weekend effect was observed by age (< 65 years: OR 1.15, 95% CI 0.97–1.35; *P* = 0.096; ≥65 years: OR 1.11, 95% CI 0.97–1.26; *P* = 0.126) or sex (male: OR 1.10, 95% CI 0.95–1.26; *P* = 0.202; female: OR 1.15, 95% CI 0.99–1.32; *P* = 0.058).

By race/ethnicity, a modest weekend effect was detected among hospitalizations involving White patients (OR 1.14, 95% CI 1.00–1.29; *P* = 0.042), whereas no significant associations were observed among hospitalizations involving Black, Hispanic, or Asian/Pacific Islander patients. By primary payer, the weekend effect was most pronounced among privately insured hospitalizations (OR 1.41, 95% CI 1.15–1.71; *P* < 0.001), with no significant differences in Medicare, Medicaid, or self-pay strata.

Hospital-level analyses suggested heterogeneity by care setting. Weekend admission was associated with higher mortality in teaching hospitals (OR 1.24, 95% CI 1.00–1.52; *P* = 0.042) and large bed-size hospitals (OR 1.26, 95% CI 1.10–1.43; *P* < 0.001). Regional variation was also observed, with a significant weekend effect in the Midwest (OR 1.32, 95% CI 1.08–1.61; *P* = 0.007) but not in the Northeast, South, or West. Rural location showed a borderline association (OR 1.27, 95% CI 0.99–1.61; *P* = 0.053).

The year-specific signal observed in 2021 should be interpreted cautiously. One possible explanation is that this period overlapped with pandemic-related strain on hospital operations, including the Delta-predominant period and the transition to the early Omicron surge in late 2021. Although we cannot directly assess COVID-19 status, staffing levels, or hospital capacity in the NIS, pandemic-era fluctuations in case volume, resource availability, and care pathways may have amplified apparent weekend–weekday differences during that year. Accordingly, we view the 2021 finding as exploratory and hypothesis-generating rather than evidence of a stable year-specific effect.

Notably, the weekend effect was not evident when stratified by the administrative marker of high-acuity PE (No: OR 1.04, 95% CI 0.86–1.25; *P* = 0.685; Yes: OR 1.06, 95% CI 0.93–1.20; *P* = 0.355). By cancer characteristics, the association was not statistically significant across major primary cancer types (e.g., lung: OR 1.19, 95% CI 0.98–1.44; *P* = 0.081; hematologic: OR 0.93, 95% CI 0.63–1.32; *P* = 0.681), and it was stronger among hospitalizations with metastatic disease (OR 1.18, 95% CI 1.03–1.34; *P* = 0.013) than among those without metastases (OR 1.06, 95% CI 0.89–1.24; *P* = 0.529). When examined by year, a significant weekend effect was observed in 2021 (OR 1.52, 95% CI 1.14–1.99; *P* = 0.003), with no significant associations in other years (Table 3).

**Table 3:** Examining the Presence of Weekend Effect in Different Subgroups.

Predictor	OR (95% CI)	*P* Value
Age		
< 65 y	1.15 (0.97–1.35)	0.096
≥ 65 y	1.11 (0.97–1.26)	0.126
Sex		
Male	1.10 (0.95–1.26)	0.202
Female	1.15 (0.99–1.32)	0.058
Race		
White	1.14 (1.00–1.29)	0.042
Black	1.16 (0.92–1.46)	0.196
Hispanic	0.92 (0.59–1.38)	0.694
Asian/Pacific Islander	0.80 (0.39–1.52)	0.507
Primary payer		
Medicare	1.09 (0.95–1.24)	0.212
Medicaid	0.82 (0.58–1.14)	0.248
Private	1.41 (1.15–1.71)	< 0.001
Self-pay	1.21 (0.62–2.22)	0.562
Hospital teaching status		
Nonteaching	1.09 (0.97–1.22)	0.160
Teaching	1.24 (1.00–1.52)	0.042
Hospital location		
Rural	1.27 (0.99–1.61)	0.053
Urban	1.10 (0.98–1.23)	0.102
Hospital bed size		
Small	0.85 (0.65–1.11)	0.237
Medium	1.02 (0.83–1.25)	0.853
Large	1.26 (1.10–1.43)	< 0.001
Region of hospital		
Northeast	1.15 (0.91–1.43)	0.239
Midwest	1.32 (1.08–1.61)	0.007
South	1.04 (0.88–1.23)	0.633
West	1.01 (0.80–1.27)	0.918
Administrative marker of high-acuity PE		
No	1.04 (0.86–1.25)	0.685
Yes	1.06 (0.93–1.20)	0.355
Primary cancer type		
Other	1.14 (0.96–1.35)	0.132
Lung	1.19 (0.98–1.44)	0.081
GI malignancies	1.22 (0.94–1.57)	0.134
Hematologic	0.93 (0.63–1.32)	0.681
Breast	1.01 (0.62–1.59)	0.959
Colorectal	1.28 (0.84–1.92)	0.236
Metastatic disease		
No	1.06 (0.89–1.24)	0.529
Yes	1.18 (1.03–1.34)	0.013
Year		
2016	0.83 (0.61–1.10)	0.204
2017	1.26 (0.97–1.62)	0.077
2018	0.94 (0.72–1.21)	0.623
2019	1.11 (0.85–1.43)	0.435
2020	1.20 (0.90–1.58)	0.197
2021	1.52 (1.14–1.99)	0.003
2022	1.17 (0.89–1.52)	0.241

Abbreviations: OR, odds ratio; CI, confidence interval. *Note: ORs represent the association between weekend (vs. weekday) admission and in-hospital mortality within each subgroup. Models were adjusted for age, sex, and year, except for the subgroup-defining variable. An OR > 1 indicates higher odds of in-hospital mortality for weekend admissions. Estimates are not shown for subgroups with insufficient sample size/unstable estimates (e.g., unweighted *n* < 30 or events < 10). The PE severity subgroup was defined using an administrative severity marker and does not correspond directly to guideline-based PE severity classification.

### Cancer-type–specific mortality and weekend effect

When stratified by primary cancer type, overall in-hospital mortality for all cancers combined was 6.1% (2,284/37,491), with higher unadjusted mortality on weekends (6.6% vs. 5.9%; *P* = 0.025) but no significant weekend effect after adjustment for age, sex, and the administrative marker of high-acuity PE (adjusted OR 1.05, 95% CI 0.95–1.17; *P* = 0.350).

Across the most common cancer subtypes, no statistically significant adjusted weekend effect was observed. For example, lung cancer had the highest event count (*N* = 8,523) and a mortality rate of 7.4%, but weekend admission was not associated with mortality after adjustment (adjusted OR 1.12, 95% CI 0.92–1.37; *P* = 0.257). Similarly, pancreatic cancer (mortality 7.8%) showed no adjusted weekend effect (adjusted OR 1.05, 95% CI 0.71–1.53; *P* = 0.816), and breast cancer (mortality 3.5%) also showed no difference (adjusted OR 0.95, 95% CI 0.59–1.53; *P* = 0.827).

In contrast, esophageal cancer was the only subtype demonstrating a significant weekend-associated increase in mortality. Among 755 esophageal cancer admissions, mortality was 10.6%, with higher weekend mortality (15.8%) compared with weekdays (9.4%) (*P* = 0.035). This association remained significant after adjustment for age, sex, and the administrative marker of high-acuity PE (adjusted OR 2.07, 95% CI 1.15–3.72; *P* = 0.015). To comply with the HCUP Data Use Agreement, cancer subtype rows containing any tabulated death cell count ≤ 10 were suppressed and are not shown in Table 4. Among the displayed cancer subtypes, esophageal cancer was the only subtype with a statistically significant weekend-associated increase in mortality after adjustment for age, sex, and the administrative marker of high-acuity PE.

**Table 4:** Cancer Subtype Analysis: Mortality and Weekend Effect in PE Patients with Different Malignancies.

Cancer type	*N*	Deaths	Mortality rate		Weekday deaths	Weekend deaths	*P* crude	OR crude	OR adjusted*	95% CI	*P* adjusted
ALL CANCERS COMBINED	37,491	2284	6.1%	1766 (5.9%)		518 (6.6%)	0.025	1.12	1.05	(0.95–1.17)	0.350
Lung cancer	8523	628	7.4%	482 (7.1%)		146 (8.3%)	0.089	1.19	1.12	(0.92–1.37)	0.257
Breast cancer	3278	115	3.5%	92 (3.5%)		23 (3.4%)	1.000	0.97	0.95	(0.59–1.53)	0.827
Colorectal cancer	2753	133	4.8%	101 (4.6%)		32 (5.7%)	0.355	1.24	1.12	(0.73–1.72)	0.612
Pancreatic cancer	2417	189	7.8%	148 (7.7%)		41 (8.3%)	0.705	1.09	1.05	(0.71–1.53)	0.816
Prostate cancer	2141	97	4.5%	79 (4.8%)		18 (3.8%)	0.417	0.78	0.72	(0.42–1.23)	0.228
Multiple myeloma	1390	52	3.7%	40 (3.8%)		12 (3.7%)	1.000	0.98	1.04	(0.53–2.05)	0.904
Uterine cancer	1253	71	5.7%	55 (5.4%)		16 (6.6%)	0.580	1.23	1.12	(0.61–2.06)	0.709
Brain cancer	1148	65	5.7%	54 (5.9%)		11 (4.8%)	0.627	0.80	0.81	(0.41–1.61)	0.551
Ovarian cancer	1141	77	6.7%	62 (6.8%)		15 (6.6%)	1.000	0.98	0.87	(0.47–1.59)	0.644
Kidney/renal cancer	1068	66	6.2%	53 (6.2%)		13 (6.0%)	1.000	0.97	0.90	(0.47–1.73)	0.751
Bladder cancer	892	51	5.7%	37 (5.2%)		14 (7.7%)	0.278	1.50	1.41	(0.73–2.72)	0.313
Esophageal cancer	755	80	10.6%	57 (9.4%)		23 (15.8%)	0.035	1.81	2.07	(1.15–3.72)	0.015
Gastric cancer	662	56	8.5%	44 (8.4%)		12 (8.6%)	1.000	1.02	1.15	(0.56–2.36)	0.709

Adjusted for age, sex, and the administrative marker of high-acuity PE.

### Outcomes and PE-related interventions by day of admission

Clinical outcomes and resource utilization differed modestly by day of admission. Length of stay among survivors was similar between weekday and weekend admissions (4.9 vs. 5.0 days; *P* = 0.242). Total hospital charges also did not differ significantly ($58,352 vs. $59,787; *P* = 0.125).

Several markers of acute critical illness were more frequent on weekends. Mechanical ventilation occurred more often among weekend admissions (4.7% vs. 3.6%; *P* < 0.001). Time to mechanical ventilation was numerically shorter on weekends (2.2 ± 4.1 vs. 2.8 ± 5.4 days), although this difference was not statistically significant (*P* = 0.088), and the proportion ventilated on hospital day 1 was similar (49.5% vs. 45.8%; *P* = 0.247). Vasopressor use was uncommon and did not significantly differ (1.4% vs. 1.1%; *P* = 0.067), nor did time to vasopressor or day-1 vasopressor use (*P* > 0.4 for both).

Use of thrombolysis was comparable between groups (4.2% weekend vs. 4.1% weekday; *P* = 0.537), with no differences in time to administration or first-hospital-day thrombolysis (*P* > 0.5 for both). Overall IVC filter placement was also similar (8.2% vs. 8.6%; *P* = 0.268), and time to placement did not differ significantly (2.7 ± 3.7 vs. 2.6 ± 3.2 days; *P* = 0.070). Among hospitalizations in which an IVC filter was placed, first-hospital-day placement was less frequent among weekend admissions than weekday admissions (14.7% vs. 18.9%; *P* = 0.016).

Consistent with Table 1, unadjusted in-hospital mortality was higher on weekends (6.6% vs. 5.9%; *P* = 0.026). Discharge disposition also differed by day of admission (overall *P* < 0.001), with fewer weekend admissions discharged home (51.9% vs. 55.9%) and higher use of skilled nursing facilities (17.2% vs. 15.0%), with corresponding shifts in other post-acute destinations (Table 5).

**Table 5:** Outcomes of Pulmonary Embolism-Related Admissions.

	Weekday (*n* = 29,687)	Weekend (*n* = 7,804)	*P* value
**Length of stay in survivors, days**	4.9	5	0.242
**Total charges**,** $**	58,352	59,787	0.125
**Mechanical ventilation**,** n (%)**	1,081 (3.6)	368 (4.7)	< 0.001
Time to mechanical ventilation, days	2.8 ± 5.4	2.2 ± 4.1	0.088
Mechanical ventilation placed on day 1, %	45.8	49.5	0.247
**Thrombolysis**,** n (%)**	1,203 (4.1)	329 (4.2)	0.537
Time to thrombolysis, days	0.9 ± 2.1	1.0 ± 1.9	0.597
Thrombolysis given on day 1, %	56.9	55.9	0.811
**Vasopressor use**,** n (%)**	327 (1.1)	106 (1.4)	0.067
Time to vasopressor, days	2.3 ± 4.7	1.8 ± 3.6	0.475
Vasopressor given on day 1, %	51.1	52.8	0.839
**IVC filter**,** n (%)**	2,546 (8.6)	638 (8.2)	0.268
Time to IVC filter, days	2.6 ± 3.2	2.7 ± 3.7	0.070
Among hospitalizations with IVC filter placement, proportion placed on hospital day 1, %	18.9	14.7	0.016
**Disposition**			< 0.001
Home, n (%)	15,599 (55.9)	3,784 (51.9)	
Short-term hospital, n (%)	660 (2.4)	207 (2.8)	
Skilled nursing facility, n (%)	4,177 (15.0)	1,250 (17.2)	
Home health care, n (%)	7,343 (26.3)	1,991 (27.3)	
Against medical advice, n (%)	129 (0.5)	52 (0.7)	

Length of stay and total charges are reported as summary estimates among survivors; corresponding P values were derived from survey-weighted analyses.

### Sequential model building explaining attenuation of the “weekend effect”

Sequential adjustment demonstrated that the apparent weekend effect was largely explained by case mix and clinical severity. In the unadjusted model, weekend admission was associated with higher mortality (OR 1.124; *P* = 0.024) and had essentially no discrimination (AUC 0.510). The association persisted with incremental adjustment for demographics, insurance, and hospital characteristics (Models 2–4; OR approximately 1.11, *P* ≈ 0.04). After adding clinical factors (Model 5), the weekend effect attenuated and became non-significant (OR 1.058; *P* = 0.302), while model discrimination improved markedly (AUC 0.755). Further adjustment for cancer type and metastatic disease did not materially change this estimate (Models 6–7). In the final model including comorbidities (Model 8), weekend admission remained non-significant (OR 1.036; *P* = 0.531) with strong overall discrimination (AUC 0.820), supporting that higher unadjusted weekend mortality reflects differences in severity and underlying risk rather than day of admission itself (Table 6).

**Table 6:** Sequential Model Building: Attenuation of the Weekend Effect With Progressive Covariate Adjustment.

Model	*N*	Events	OR	CI lower	CI upper	OR (95% CI)	*P* value	AIC	AUC
Model 1: Unadjusted	37,481	2284	1.12	1.02	1.24	1.124 (1.016–1.244)	0.024	17205.7	0.510
Model 2: + Demographics	36,492	2214	1.11	1.00	1.23	1.112 (1.004–1.233)	0.043	16664.2	0.546
Model 3: + Insurance	36,456	2212	1.12	1.01	1.24	1.116 (1.007–1.237)	0.037	16634.3	0.556
Model 4: + Hospital	36,456	2212	1.11	1.00	1.24	1.114 (1.005–1.235)	0.040	16605.8	0.569
Model 5: + Clinical	36,408	2210	1.06	0.95	1.18	1.058 (0.951–1.177)	0.302	14667.7	0.755
Model 6: + Cancer type	36,408	2210	1.06	0.95	1.18	1.058 (0.951–1.178)	0.302	14594.2	0.766
Model 7: + Metastasis	36,408	2210	1.06	0.95	1.18	1.058 (0.950–1.178)	0.304	14434.2	0.782
Model 8: + Comorbidities (Final)	36,408	2210	1.04	0.93	1.16	1.036 (0.928–1.156)	0.531	13694.5	0.820

## Discussion

In this nationally representative cohort of U.S. hospitalizations for cancer-associated pulmonary embolism (PE) from 2016 to 2022, weekend admission was associated with higher unadjusted in-hospital mortality, but was not independently associated with mortality after multivariable adjustment. Sequential model building further demonstrated that the crude weekend association attenuated after incorporation of clinical covariates while model discrimination improved, supporting the interpretation that the apparent “weekend effect” primarily reflects differences in case mix and illness severity at presentation rather than weekend timing itself.

### Relationship to prior “weekend effect” literature in PE and acute care

Our findings align with and extend prior work. In a seminal NIS-based analysis of PE admissions, Nanchal and colleagues reported higher mortality for weekend admissions and later timing of some procedures, including IVC filter placement^6^. In contrast, in this contemporary cancer-associated PE cohort (2016–2022), weekend admission was not independently associated with in-hospital mortality after adjustment for PE severity, metastatic burden, and comorbidity. This pattern is consistent with broader weekend-effect literature showing that excess weekend mortality often attenuates after adjustment for illness severity and case mix^10^. Although weekend operational factors may still influence specific care processes, our results suggest that, at the hospitalization level, measured clinical risk is the main driver of mortality differences by day of admission.

### Why cancer-associated PE is distinct

Cancer-associated PE represents a biologically and clinically distinct phenotype characterized by higher baseline vulnerability and competing risks^11,12^. Prior inpatient analyses have shown that concurrent cancer is associated with higher mortality, longer length of stay, and greater costs among PE hospitalizations^3,13^. In parallel, population-level data demonstrate substantial PE-related mortality burden in people with cancer, underscoring the importance of prevention and optimized acute management^14^.

In our fully adjusted mortality model, the administrative marker of high-acuity PE and metastatic disease were dominant predictors of death, and several comorbidity domains plausibly reflecting frailty and acute physiologic derangement (e.g., fluid/electrolyte disorders, arrhythmias, coagulopathy, weight loss/cachexia) were independently associated with mortality. Taken together, these findings support a cancer-specific interpretation: weekend admission status is less informative than severity and oncologic/physiologic vulnerability, which should remain the principal focus of risk stratification and early escalation decisions in hospitalizations for cancer-associated PE^13^.

### Process-of-care signals on weekends: descriptive and context-dependent

We observed higher rates of mechanical ventilation among weekend admissions, while thrombolysis rates and overall IVC filter use were similar between groups. Among hospitalizations in which an IVC filter was placed, first-hospital-day placement was less frequent on weekends^2^. This finding should be interpreted cautiously. Unlike immediate anticoagulation, thrombolysis, or hemodynamic stabilization, IVC filter placement is not uniformly a time-critical intervention and may reasonably be deferred in selected cases, particularly when weekend procedural services are prioritized for emergent indications. Accordingly, lower first-hospital-day IVC filter placement on weekends may reflect differences in procedural triage, consultation workflow, scheduling, or local practice patterns rather than a delay in care or a deficiency in quality^15^. Because administrative data do not capture the indication for filter placement, contraindications to anticoagulation, bleeding events, hemodynamics, imaging findings, or PE response-team activation, this result should be interpreted as a difference in timing pattern rather than evidence of under-treatment^16–18^.

### Heterogeneity: subgroup and cancer-type findings should be treated as exploratory

Subgroup analyses suggested heterogeneity in the crude weekend association across payer, hospital type/size, region, metastatic status, and year, particularly 2021. However, because these models were intentionally parsimonious and did not include the full covariate set from the primary model, these findings should be considered exploratory and vulnerable to residual confounding^19,20^. Future work should test formal interactions within fully adjusted models and validate these signals in datasets with richer clinical detail and time-stamped care processes.

In cancer-type–stratified analyses, esophageal cancer was the only subtype with a significant adjusted weekend-associated increase in mortality. This finding should be interpreted cautiously given multiple comparisons and limited adjustment in the stratified model. Still, the signal may be clinically plausible because patients with esophageal cancer often have impaired physiologic reserve related to dysphagia, anorexia, cachexia, and malnutrition^21,22^. We therefore consider this a hypothesis-generating finding that warrants confirmation in independent cohorts.

### Generalizability

Because the NIS is a nationally representative sample of U.S. community-hospital discharges, our estimates are broadly generalizable to inpatient care for cancer-associated PE in U.S. community hospitals. However, results should not be extrapolated to non-inpatient settings, to longitudinal patient-level outcomes after discharge, or to questions requiring patient-level follow-up across multiple hospitalizations, because the NIS is discharge-based and does not permit tracking of individuals over time.

### Limitations

As with all administrative database studies, diagnoses, comorbidities, and severity constructs depend on coding and may be misclassified. The dataset lacks granular physiologic and imaging variables, anticoagulation details, contraindications, bleeding events, and goals-of-care intensity, leaving potential for residual confounding. Weekend admission represents timing of admission rather than onset of decompensation and does not directly measure staffing, specialist availability, or time-to-treatment processes. In particular, the NIS does not capture the indication for IVC filter placement, contraindications to anticoagulation, procedural urgency, or whether lower first-hospital-day filter placement on weekends was offset by later in-hospital placement; therefore, differences in first-hospital-day IVC filter use should not be interpreted as a direct measure of care quality. Finally, subgroup and cancer-type analyses may be underpowered for less common cancers, and multiple comparisons increase the likelihood of chance findings. We additionally performed sensitivity analyses using administrative proxies of goals-of-care status, including palliative care encounter and DNR codes, and the weekend-effect estimate remained materially unchanged. However, these codes may incompletely capture goals-of-care discussions and do not establish temporality relative to admission or clinical deterioration. In addition, our PE severity construct was an administrative severity marker based on coded diagnoses/procedures and should not be interpreted as equivalent to guideline-based PE severity classification. Because the NIS does not provide clinical chronology for these coded events, we could not determine whether respiratory failure, cardiogenic shock, or cardiac arrest reflected the same episode of decompensation or the exact temporality relative to admission.

## Conclusions

In U.S. hospitalizations for cancer-associated PE (2016–2022), weekend admission was associated with slightly higher unadjusted mortality but was not an independent predictor of in-hospital mortality after accounting for severity, metastatic burden, and comorbidity profile. These findings suggest that the crude weekend mortality difference largely reflects risk concentration and case mix. Future work should focus on validating subgroup signals (including esophageal cancer) and evaluating time-sensitive care processes using clinically granular datasets capable of disentangling severity, treatment pathways, and operational factors.

### Supplementary Information

eMethods; eTable S1: ICD-10-CM codes used to define the acute PE cohort (principal diagnosis); eTable S2: ICD-10-CM definitions for malignancy, metastasis, and cancer-type groupings; eTable S3: ICD-10-PCS procedure codes for PE-related interventions and timing definitions; eTable S4: Key HCUP NIS variables used (exposure, outcomes, and survey design). eTable S5. Sensitivity analyses of the association between weekend admission and in-hospital mortality after incorporation of palliative care encounter and do-not-resuscitate (DNR) status.

## Supplementary Information

Below is the link to the electronic supplementary material.

Supplementary Material 1

## Data Availability

The data analyzed in this study were obtained from the Healthcare Cost and Utilization Project (HCUP) National Inpatient Sample (NIS), 2016–2022. The HCUP NIS data are available from the HCUP Central Distributor under a data use agreement (DUA) and are not freely publicly downloadable. Researchers may obtain access by completing HCUP DUA training and purchasing the dataset through the HCUP Central Distributor.(see HCUP NIS Overview: https://hcup-us.ahrq.gov/nisoverview.jsp; HCUP NIS purchase and download platform: https://cdors.ahrq.gov/databases; HCUP Central Distributor Data Use Info: https://cdors.ahrq.gov/data-use-info; HCUP DUA Training: https://hcup-us.ahrq.gov/tech_assist/dua.jsp).The authors are not permitted to share the raw NIS data due to the HCUP DUA restrictions. Analytic code can be made available from the corresponding author upon reasonable request.

## Abbreviations

AUC/C-statistic: Area under the receiver operating characteristic curve
CI: Confidence interval
HCUP: Healthcare Cost and Utilization Project
IVC: Inferior vena cava
NIS: National Inpatient Sample
OR: Odds ratio
PE: Pulmonary embolism
STROBE: Strengthening the Reporting of Observational Studies in Epidemiology
