# Supplementary material for: Weekend admission and outcomes in cancer-associated pulmonary embolism: a cross-sectional national inpatient sample study, 2016–2022
Source: Sci Rep. 2026 May 22;16:23392. doi: 10.1038/s41598-026-50298-4 (PMC13408675; doi:10.1038/s41598-026-50298-4)
Supplement: Supplementary file 1 — Supplementary Material 1 [file 41598_2026_50298_MOESM1_ESM.docx]

**Supplementary — eMethods and eTables**

**eMethods 1. Data Source and Study Design**

We conducted a retrospective cohort study using the Healthcare Cost and Utilization Project (HCUP) National Inpatient Sample (NIS) for 2016–2022, which contains discharge-level data and is designed to generate national estimates when analyzed with the provided discharge weights and complex survey design variables. Beginning in 2016, the NIS is a calendar-year file with diagnoses and procedures coded exclusively using ICD-10-CM/PCS.

**eMethods 2. Study Population**

**Inclusion criteria**

1. Adult hospitalizations (age ≥18 years).
2. A principal diagnosis of acute pulmonary embolism (PE) using ICD-10-CM codes listed in eTable S1.
3. Evidence of active malignancy during the hospitalization, defined by the presence of any ICD-10-CM malignant neoplasm code C00–C96 in any diagnosis field (excluding *in situ* neoplasms and history codes), per eTable S2.

**Exclusion criteria**

1. Chronic PE (ICD-10-CM I27.82) and traumatic embolism codes (e.g., T79.0xxA, T79.1xxA) to restrict the cohort to acute PE presentations (eTable S1).
2. Records with missing weekend indicator (AWEEKEND missing/invalid) were excluded from weekend-effect analyses.

**eMethods 3. Exposure: Weekend Admission**

The exposure was weekend admission using the NIS variable AWEEKEND, which is derived from the admission date. Weekend admissions are defined as Saturday and Sunday in HCUP documentation, with missing/invalid flags when admission date is missing/invalid.

**eMethods 4. Outcomes**

**Primary outcome**

- **In-hospital mortality**, defined by NIS variable **DIED** (death during hospitalization).

**Secondary outcomes**

- Length of stay (LOS).
- Total hospital charges (TOTCHG).
- Discharge disposition (DISPUNIFORM/DISPUNIFORM2 as applicable by year; harmonized to uniform categories).
- PE-related organ support and interventions: mechanical ventilation, vasopressor administration, thrombolysis, IVC filter placement, and their timing using PRDAYn variables (definitions and codes in eTable S3; timing approach in eMethods 6).

**eMethods 5. Covariates**

We adjusted for:

- **Demographics:** age group, sex, race/ethnicity.
- **Socioeconomic/payer:** primary expected payer.
- **Hospital characteristics:** region, bed size, teaching status (as defined in NIS).
- **Clinical factors:**
  - Metastatic disease (yes/no) defined by metastatic ICD-10-CM families C77–C79 and C80.0 (disseminated malignant neoplasm) present in any diagnosis field (eTable S2).
  - Comorbidities based on HCUP Elixhauser Comorbidity Software Refined for ICD-10-CM.

**Primary cancer type classification.** Primary cancer type was assigned from diagnosis fields using ICD-10-CM malignant neoplasm site codes (C00–C76) and hematologic codes (C81–C96), grouped into prespecified categories (e.g., lung, breast, colorectal, prostate, pancreas, esophagus, hematologic), per eTable S2. When multiple cancer site codes were present, a hierarchical rule was applied to prioritize (1) solid organ site codes over (2) nonspecific codes (C80.1) and to classify “hematologic” if any C81–C96 was present.

**eMethods 6. Procedure Identification and Timing**

Procedures were identified using ICD-10-PCS procedure codes (eTable S3). Procedure timing was defined using PRDAYn variables:

- Day 0 = procedure performed on the day of admission.
- “Early” procedure = PRDAYn = 0 (admission day).
- Missing/invalid/inconsistent PRDAYn were handled per HCUP guidance: PRDAYn can be missing (.), invalid (.A/.B), or inconsistent (.C) depending on availability/validity of admission date and procedure date/day information. Timing analyses treated non-numeric PRDAYn flags as missing and did not impute timing.

**eMethods 7. Statistical Analysis**

All analyses accounted for NIS complex sampling using discharge weights and stratification/clustering:

- We used TRENDWT for pooled multi-year analyses spanning 2016–2022 (merged by year and hospital identifier as recommended by HCUP).
- Survey design used: weights = TRENDWT, strata = NIS_STRATUM, cluster/PSU = HOSP_NIS.

**Critical multi-year pooling note:** HCUP specifies that HOSP_NIS is reassigned each year, so it cannot link hospitals across years. Therefore, for pooled analyses, we treated clusters as year-specific by creating a unique PSU such as PSU_YEAR = interaction(YEAR, HOSP_NIS) to prevent inadvertent cross-year clustering.

In R, models were fit with the *survey* package using svydesign(…, nest=TRUE) and svyglm() for logistic regression; nest=TRUE enforces appropriate nesting of PSUs within strata.

**eMethods 8. Inflation Adjustment**

Charges (TOTCHG) were reported in constant 2022 U.S. dollars by inflating/deflating year-specific charges using the Consumer Price Index (CPI-U).

**eTable S1.** ICD-10-CM Codes Used to Define the Acute PE Cohort (Principal Diagnosis)

| **Construct** | **ICD-10-CM Codes** | **Notes** |
| --- | --- | --- |
| Acute PE with acute cor pulmonale | I26.01, I26.02, I26.09 | Included if present as principal diagnosis |
| Acute PE without acute cor pulmonale | I26.90, I26.92, I26.93, I26.94, I26.99 | Included if present as principal diagnosis |
| Exclusions (non-acute PE entities) | I27.82 | Chronic pulmonary embolism |
| Exclusions (traumatic embolism) | T79.0xxA, T79.1xxA | Traumatic air/fat embolism (excluded to restrict to acute PE) |

**eTable S2.** ICD-10-CM Definitions for Malignancy, Metastasis, and Cancer-Type Groupings

**A) Active Malignancy (Required)**

| **Construct** | **ICD-10-CM Families Included** | **ICD-10-CM Families Excluded** | **Notes** |
| --- | --- | --- | --- |
| Active malignancy during index admission | C00–C96 | D00–D09; Z85.* | Excludes in situ and history of malignancy codes |

**B) Metastatic Disease (Covariate)**

| **Construct** | **ICD-10-CM Families Included** | **Notes** |
| --- | --- | --- |
| Metastatic disease | C77.*, C78.*, C79.*, C80.0 | Secondary malignant neoplasms and disseminated malignancy |

**C) Primary Cancer Type Grouping**

| **Cancer Category** | **ICD-10-CM Families/Codes** | **Notes (Assignment)** |
| --- | --- | --- |
| Lung | C34.* | Site-based grouping |
| Breast | C50.* | Site-based grouping |
| Colorectal | C18.*, C19, C20 | Site-based grouping |
| Pancreas | C25.* | Site-based grouping |
| Esophagus | C15.* | Site-based grouping |
| Prostate | C61 | Site-based grouping |
| Hematologic malignancy | C81–C96 | Lymphoma/leukemia/myeloma group |
| Other solid tumors | Remaining C00–C76 not above | Site-based remainder |
| Unspecified primary | C80.1 | Kept as “other/unspecified” unless a specific site code is present |

***Note:*** Specific site codes supersede C80.1; “hematologic” assigned if C81–C96 present and no specific solid-site category is assigned.

**eTable S3.** ICD-10-PCS Procedure Codes for PE-Related Interventions and Timing Definitions

**A) Procedures (Receipt)**

| **Intervention** | **ICD-10-PCS Codes** | **Descriptor (PCS Intent)** |
| --- | --- | --- |
| Mechanical ventilation, <24 h | 5A1935Z | Respiratory ventilation, <24 consecutive hours |
| Mechanical ventilation, 24–96 h | 5A1945Z | Respiratory ventilation, 24–96 consecutive hours |
| Mechanical ventilation, >96 h | 5A1955Z | Respiratory ventilation, >96 consecutive hours |
| Vasopressor administration (venous) | 3E033XZ, 3E043XZ | Introduction of vasopressor into peripheral vein / central vein, percutaneous approach |
| Systemic thrombolysis (venous) | 3E03317, 3E04317 | Introduction of thrombolytic into peripheral vein / central vein, percutaneous approach |
| IVC filter placement | 06H03DZ, 06H00DZ | Insertion of intraluminal device into IVC (percutaneous / open approach) |

**B) Timing (Early vs. Not Early)**

| **Timing Variable** | **Definition** | **Rule Used** |
| --- | --- | --- |
| Procedure day | PRDAYn | “Number of days from admission to procedure n” |
| Early (“hospital day 1”) | PRDAYn = 0 | Defined as performed on day of admission |
| Missing/invalid PRDAYn | Non-numeric PRDAYn flags or missing | Treated as missing for timing analyses; not imputed |

**eTable S4.** Key HCUP NIS Variables Used (Exposure, Outcomes, and Survey Design)

| **Variable** | **Meaning** | **Coding / Usage in Analysis** |
| --- | --- | --- |
| AWEEKEND | Weekend admission | 0 = weekday; 1 = weekend |
| DIED | In-hospital mortality | 0 = survived; 1 = died |
| LOS | Length of stay | Continuous (days) |
| TOTCHG | Total hospital charges | Continuous (USD, nominal unless CPI-adjusted) |
| PRDAYn | Days from admission to procedure n | Used for time-to-procedure and “day 1” definitions |
| DISCWT | Discharge weight | Used to generate national estimates |
| NIS_STRATUM | Stratum | Used for variance estimation |
| HOSP_NIS | Hospital identifier (within-year) | Used as PSU/cluster; for pooled years, treat as year-specific cluster |

**eTable S5.** Sensitivity Analyses Addressing Goals-of-Care Status in the Association Between Weekend Admission and In-Hospital Mortality

| **Model** | ***N*** | **OR for Weekend Admission** | **95% CI** | ***P* Value** |
| --- | --- | --- | --- | --- |
| Original fully adjusted model | 36,408 | 1.043 | 0.932–1.168 | 0.465 |
| Fully adjusted + palliative/DNR combined | 36,408 | 1.018 | 0.902–1.148 | 0.776 |
| Fully adjusted + palliative + DNR as separate covariates | 36,408 | 0.992 | 0.876–1.122 | 0.894 |
| Excluding hospitalizations with palliative care and/or DNR codes | 27,479 | 0.987 | 0.802–1.215 | 0.903 |

***Abbreviations:*** OR, odds ratio; CI, confidence interval; DNR, do-not-resuscitate. All models use survey-weighted logistic regression with the NIS complex sampling design.
